# Linac primary barrier transmission for concrete: Monte Carlo calculations

**DOI:** 10.1002/acm2.13847

**Published:** 2022-12-05

**Authors:** Patrick N. McDermott

**Affiliations:** ^1^ Radiation Oncology Department Beaumont Health System Royal Oak Michigan USA

**Keywords:** Linac, primary barrier, radiation safety, shielding, TVL

## Abstract

Recent publications have called into question the accuracy of reference tenth‐value layer (TVL) data cited in official reports for linac primary concrete barriers. Doubts have arisen based on both experimental and theoretical evidence. Most of the standard reference TVL values trace back to a publication that appeared in 1984 that used beam spectra that are not representative of modern linacs. This study reports a new set of TVL data for concrete based on modern linac beam spectra and a definition of the barrier transmission that is consistent with its use in shielding calculations. TVL values have been computed for concrete using Monte Carlo simulation for beam energies of 4, 6, 10, 15, and 18 MV. The barrier transmission depends on the field size at the barrier and the distance from the distal surface of the barrier to the point of observation. The TVL values reported here lead to barrier transmission values that are up to a factor of 4 larger than those in official reports. The air kerma rate beyond the barrier does not obey an inverse square law as the barrier now acts like a new (non‐point) source of radiation. For distance greater than 0.3 m from the distal side of the barrier, inverse square predictions of the air kerma rate are low by up to a factor of 2. The average energy of the transmitted photons declines rapidly for all beam energies with increasing barrier thickness up to a thickness of about 50 cm and then slowly increases with increasing thickness.

## 1 INTRODUCTION

The International Atomic Energy Agency (IAEA) Directory of Radiotherapy Centres database reports that there are currently over 18,000 megavoltage radiation therapy units worldwide.^1^ For linac barrier construction, concrete is usually the material of choice “since it is the least expensive.”^2^ Two recent publications have cast doubt on the accuracy of primary barrier concrete tenth‐value layer (TVL) data listed in official references such as the National Council on Radiation Protection and Measurements Report No. 151 (NCRP151), the Institute of Physics and Engineering in Medicine Report 75 (IPEM75), and the International Atomic Energy Agency Report 47 (IAEA47).^3^, ^4^, ^5^, ^6^ The concrete TVL data for 4–18 MV are identical in NCRP151 and IPEM75. Measurements have shown that the TVL data from these publications sometimes underpredicts air kerma rates by up to a factor of 3.^3^ This may be the reason that the textbook by Martin and McGinley recommends a multiplicative “margin” of a factor of two or three.^7^ In addition to this, more recent determinations of TVL data predict barrier transmission values that are higher than NCRP151 by as much as a factor of 4.^3^ Most of the TVL data in the official references traces back to a common origin: a 1984 publication by Nelson and LaRiviere (NL).^8^ The TVL values in the NL publication are based on linac spectra that do not accurately represent contemporary linacs. In addition, the NL calculations were based on the use of buildup factors rather than full Monte Carlo calculations.

The barrier transmission, *B*, depends on the beam energy, the density of the concrete, the field size at the barrier, any extra material on or inside the barrier, and the distance of measurement from the distal barrier surface. Comparisons between measured and predicted barrier transmission do not usually account for all of these variables. This may be the reason that widely different barrier transmission values have been measured for the same barrier thickness, composition, and energy. These confounding factors can mask discrepancies between predicted and measured values. Density variations can lead to under or overestimates of *B*. Reference values of TVL are based on standard density concrete (2.35 g/cm^3^). It is reported that concrete density can vary between 2.09 and 2.50 g/cm^3^.^9^ This can lead to variations as large as a factor of about 3–4 in *B* compared to standard density.^3^ This underscores the importance of the usual recommendation that concrete density be tested and documented.

There is almost always additional material within or on barriers, besides concrete, including steel rebar, laser mounting plates, a masonry façade, dry wall, roofing material, etc. These additional materials are rarely accounted for and will lead to measured values of *B* that are lower than for the concrete barrier alone. These materials can have a surprisingly large effect if they are on the outside of the barrier (see Section 3.2) where the emerging beam energy is quite low.

Previous determinations of TVL data for concrete primary barriers have been made by NL, Jaradat and Biggs (JB), Karoui and Kharrati (K2), and Rijken, Town, and Healy (RTH).^4^, ^10^, ^11^ Various references quote TVL data in different formats. The IAEA47 quotes single values of the TVL for each energy that “are approximate values based on large attenuation.” ^6^ NCRP151, IPEM75, and K2 quote values of TVL_1_ and TVL*_e_*. TVL_1_ is the first tenth value thickness, and TVL*_e_* is the “equilibrium value.” Values of *B* are computed from the following formula:

(1) $$B=10^{-1}\;\times \;10^{-\left(t-{\mathrm{TVL}}_{1}\right)/{\mathrm{TVL}}_{e}}\;\mathrm{for}\;t>\mathrm{TV}L_{1},$$

where *t* is the thickness of the barrier. Other references, such as JB and NL, quote values of TVL_1_, TVL_2_, and TVL_3_. In this case, values of *B* are computed from the following formulas:

(2) $$\begin{array}{c} \begin{array}{cc} B=10^{-\frac{t}{{\mathrm{TVL}}_{1}}} & \mathrm{for}\;t<{\mathrm{TVL}}_{1}\\ B=10^{-1}\times 10^{-\frac{t-{\mathrm{TVL}}_{1}}{{\mathrm{TVL}}_{2}}} & {\mathrm{for}\;\mathrm{TVL}}_{1}<t<{\mathrm{TVL}}_{1}+{\mathrm{TVL}}_{2}\\ B=10^{-2}\times 10^{-\frac{t-{\mathrm{TVL}}_{1}-{\mathrm{TVL}}_{2}}{{\mathrm{TVL}}_{3}}} & \mathrm{for}\;t>{\mathrm{TVL}}_{1}+{\mathrm{TVL}}_{2}. \end{array} \end{array}$$

The values of the parameters TVL_1–3_ in Equation (2) and TVL_1_ and TVL*_e_* in Equation (1) should be based on best fits to calculated values of *B*. For this reason, the value of TVL_1_ in Equation (1) is not expected to be precisely equal to the value of TVL_1_ in Equation (2) even for the same barrier transmission curve. Small differences in TVL can lead to large differences in *B*, as the TVL values appear in an exponent.

This study has not considered the barrier transmission of flattening filter free beams (FFF). That topic has been addressed by McDermott et al. by comparing measurements of the transmission of FFF beams to flattened beams.^12^ This study also does not address barrier transmission for heavy concrete.

### 1.1 Purpose

The purpose of this investigation is to calculate new barrier transmission factors for concrete using Monte Carlo simulations along with a mathematically consistent definition of *B*, and to compare them with previously calculated values. An attempt is made to avoid some of the limitations of previous determinations as described in Section 1.2. New values of TVL are computed from fits to barrier transmission data using Equations (1) and (2). A secondary goal is to provide additional physical insight into the broad beam barrier penetration problem. In addition, a description is provided of the energy distribution of the transmitted beam, the dependence of the instantaneous air kerma rate (AKR) with distance from the distal surface of the barrier, and the field size dependence of *B*.

### 1.2 Critique of previous TVL determinations

Most, if not all, previous calculations of barrier transmission are mathematically inconsistent with the use of this quantity in standard shielding calculations. The standard NCRP151 equation for primary barrier calculation of the weekly air kerma is *P* = *B* WUT/*d* ^2^, where “*W* is the workload or photon‐absorbed dose delivered at 1 m from the X‐ray target per week (Gy/week), *U* is the use factor, *T* is the occupancy factor, and *d* is the distance from the X‐ray source to the point of interest (in meters).” ^2^ We submit that the proper definition of barrier transmission is

(3) $$B=\frac{{\dot{K}}_{a}}{{\dot{D}}_{0}}{\left(\frac{d}{1\;m}\right)}^{2},$$

where ${\dot{K}}_{a}$ is the AKR measured in Sv/h (= Gy/h) at a point 0.3 m beyond the distal face of the barrier on the central axis, *d* is the distance from the source (X‐ray target) in meters, and ${\dot{D}}_{0}$ is the instantaneous absorbed dose rate (workload in Gy/h) in a large water phantom on the central axis at a depth of *d*_max_ and at a distance of 1 m from the source for a 10 × 10 cm^2^ field. ${\dot{D}}_{0}$ can be easily tied directly to the “dose rate” of the linac in MU/min via TG51 beam calibration. The “dose rate” can be read directly from the linac console. The transmission will depend on the field size at the barrier. We ignore the head scatter factor *S_c_*. For a 40 × 40 cm^2^ field size, this is about 10% or less. The JB calculations are based on the ratio of energy fluence with and without the barrier present. This ignores changes in the beam spectrum as a result of passage through the barrier, and it is inconsistent with Equation (3). As shown in Section 3.2, the emergent beam spectrum is radically different from the incident beam spectrum. The calculations by NL and K2 are based on the ratio of air kerma with and without the barrier present. These calculations are also inconsistent with Equation (3).

TVL values listed in NCRP151, IPEM75, and Martin and McGinley (except 10 MV) can all be traced back to the 1984 Nelson and LaRiviere (NL) paper.^8^ NL have calculated values of *B* for beams with energies of 6, 10, and 25 MV. Values of the TVL quoted by NCRP151 for 4 MV are based on an extrapolation, most likely from the values for 6 and 10 MV. It appears as if the NCRP151 values for 15 and 18 MV are based on interpolations between the NL values for 10 and 25 MV.

The buildup factors used by NL have been taken from a 1966 publication by Truby, based on an isotropic source in an infinite medium.^13^ RTH have shown that Monte Carlo calculations of *B* for a 10 MV beam differ significantly from those calculated using the Truby buildup factors for concrete thickness greater than 180 cm. These authors state that the Truby buildup factors are only applicable up to a depth corresponding to 20 mean free path lengths (which is, of course, energy dependent). The beam spectra used by NL do not accurately represent contemporary linacs. As an example, the average photon energy of the NL 10 MV beam is 2.6 MeV. The average energy of the 10 MV linac spectra computed by Sheikh‐Bagheri and Rogers (see Section 2.1) is 3.3 MeV.^14^ The ratio of narrow beam monoenergetic transmission for these two energies traversing 1.5 m of concrete is 4.8.

The JB calculations are only carried out to a depth of 76.5 cm for Co‐60 and 151.5 cm for 18 MV, respectively. For 18 MV, this corresponds to *B* ∼ 10^−3^. Clinically realistic values of *B* are on the order of 10^−5^, and thus, JB TVL data for clinically relevant depths are based on extrapolation.

## 2 METHODS

### 2.1 The Monte Carlo code

The MC code, hereafter referred to as MCBT, is a dedicated program written using the Mathematica software (version 12.2.0.0).^15^ It is designed specifically for barrier penetration calculations. It is a modified version of the “MCLS” code described by McDermott for linac skyshine calculations.^16^ Skyshine calculations require photon transport through a primary concrete roof barrier before photons can be scattered to ground level. The advantage of a dedicated code is that it allows complete and total control over all aspects of the calculation. The disadvantage is the need for code validation. Tests of the MCBT code are described below, but first we describe the code. This description follows the recommendations of TG‐268, “Improved reporting of Monte Carlo radiation transport studies.”^17^

The code uses a divergent beam of square cross section of adjustable size perpendicularly incident on a concrete slab of variable thickness. The photon fluence is uniform across the square field. The lateral distance from the central beam axis to the edge of the slab is 10 m. The distance from the radiation source to the distal barrier surface is nominally 6.0 m. Photons are discarded if they reach a lateral distance of 10 m or more from the central axis, or if they are reflected back into the vault. Attenuation by air is ignored. This is expected to be on the order of 2%–4%. There is no rebar in the concrete. The concrete composition is based on White–Grodstein (1957) and McGinnies (1959).^18^, ^19^ Table 1 gives the weight fraction for each element in the mix. The mass attenuation coefficients were taken from the NIST XCOM database.

**TABLE 1:** Concrete composition (weight fraction)

Element	H	O	Na	Mg	Al	Si	S	K	Ca	Fe
Fraction	0.0056	0.4983	0.0171	0.0024	0.0456	0.3158	0.0012	0.0192	0.0826	0.0122

Photons are “killed” when their energy falls below 10 keV. The code does not include charged particle transport; thus, there is no bremsstrahlung production in the barrier. Pair production is included, but pair annihilation photons are assumed created “on the spot” and are emitted in random directions with a 180° angle between each photon. It is assumed that the energy spectrum of the photons does not vary off axis or with field size.

The differential fluence spectra of the incident photon beams are taken from Sheikh‐Bagheri and Rogers, for 4, 6, 10, 15, and 18 MV Varian linac 10 × 10 cm^2^ beams.^14^ The only Elekta linac spectra reported in this reference are for 6 and 25 MV. The Elekta 6 MV beam spectrum has been included in this study. The Elekta spectrum has a slightly higher beam energy. The percent depth dose (field size 10 × 10 cm^2^) at a depth of 10 cm is 67.4% for Elekta and 65.9% for Varian. The Sheikh‐Bagheri and Rogers spectra reproduce measured depth–dose curves very accurately. Table 2 shows a comparison of the calculated percent depth dose from Sheikh‐Bagheri and Rogers with measured data from Varian TrueBeam linacs^20^ and with the Elekta specification for a Versa HD 6 MV beam.^*^ The second column of Table 2 shows the measured data (or the Elekta specification), and the third column shows the values quoted by Sheikh‐Bagheri and Rogers. The Sheikh‐Bagheri and Rogers spectra show significant beam softening off axis (except for 4 MV). This has not been included in the Monte Carlo calculations. Table 3 lists the average energy of each beam and the dose per photon at a distance of 100 cm from the source and at *d*_max_ on the central axis for a 10 × 10 cm^2^ field. The data in the last column of Table 3 has been used to compute ${\dot{D}}_{0}$ in Equation (3).

**TABLE 2:** Comparison of calculated versus measured percent depth dose

Energy	PDD(10)^a^	%*dd_c_* (10)^b^
6	66.2 ± 0.3	65.9 ± 0.3
6^c^	67.5 ± 1	67.4 ± 0.3
10	73.5 ± 0.1	73.4 ± 0.3
15	76.6 ± 0.1	76.4 ± 0.3

^a^ Measured.

^b^ Calculated.

^c^ Elekta Versa HD specification.

**TABLE 3:** Incident beam parameters^a^

Energy (MV)	Average energy (MeV)^a^	Dose/photon (Gy)
4	1.52	(6.031 ± 0.058) E − 14
6	1.79	(6.497 ± 0.025) E − 14
6^b^	2.01	(7.403 ± 0.025) E − 14
10	3.20	(1.028 ± 0.036) E − 13
15	3.88	(1.066 ± 0.045) E − 13
18	4.89	(1.288 ± 0.056) E − 13

^a^ Computed with MCBT code.

^b^ Elekta beam spectrum.

There is a series of “detectors” arrayed along the central axis. Each of these is a thin circular cylinder with a radius *r_ic_*. The principal detector resides at a distance of 0.3 m from the distal surface of the barrier. The air kerma is tallied in this detector, and that value is used in Equation (3) to calculate *B*. The air kerma is computed from the fluence tallied in the detectors along with air kerma conversion factors to convert fluence to kerma. The statistical uncertainty in the fluence is computed using the “history‐by‐history” method.^21^ The other axial detectors are intended to elucidate the dependence of the air kerma as a function of distance beyond the distal surface of the barrier. In addition, there is a series of coaxial detectors, bounded by an inner and an outer square, in a plane at a distance of 0.3 m from the distal surface. Each of these detectors has a width of 15 cm for the purpose of monitoring the cross‐plane profile.

It is desirable for the principle detector to have as large a radius as possible to increase the number of photons passing through it and thus decrease the statistical uncertainty. The number of photons passing through the MCBT principal detector is approximately equal to

(4) $$N_{\mathrm{ic}}∼N_{0}B\frac{A_{\mathrm{ic}}}{A_{b}},$$

where *N*_0_ is the number of photon histories, *B* is the transmission, *A_ic_* is the cross‐sectional area of the cylindrical detector, and *A_b_* is the cross‐sectional area of the beam projected to the position of the detector.^†^ The value of *B* can be as small as 10^−6^, and therefore, *A_ic_* should be as large as possible. On the other hand, the fluence should not vary significantly across the detector. When the distal surface is at a distance of 6 m from the target, the side length of a field that is 40 × 40 cm^2^ at isocenter diverges to 240 cm. Under these circumstances, *r_ic_* has been set to 50 cm. Cross beam profiles at a source distance of 6.3 m show little variation over this distance. The value of *r_ic_* has been adjusted up or down as needed for other field sizes at the barrier. As many as 3 × 10^8^ histories have been followed for the thickest barriers. This results in a statistical error in the transmission (1 SD) of about 4%.

Calculations of *B* should be extended to thicknesses that are clinically realistic, otherwise TVL determinations are based on extrapolations. The required value of *B* can be assessed as follows. Assume that the inner surface of the barrier is at a distance of 3 m from isocenter. This is approximately the minimum distance allowed by Varian and Elekta.^‡^ ^,^ ^#^ The total distance of the inner surface will then be 4 m from the target. Assume that the total distance to the detector is *d* = 6.3 m from the target (the barrier is roughly 2 m thick). Assume an uncontrolled area with a weekly limit of *P* = 2 × 10^−5^ Sv, and occupancy *T* = 1, *U* = 0.25, *W* = 10^3^ Gy/week. Under these circumstances, *B* = 3.2 × 10^−6^. This result is a very conservative, energy independent, estimate of the required value of *B*. Calculations of clinically relevant TVL values based on larger *B* values are extrapolations. Such extrapolations may be valid to the extent that the beam energy does not change as the thickness increases beyond the points used in the extrapolation. The practical difficulty with MC calculations for such small values of *B* is that it requires a very large number of photon histories (and hence large CPU time) to obtain statistically meaningful results. We have used up to 3 × 10^8^ histories. The laptop computer used to perform these calculations can execute approximately 5 × 10^4^ histories per minute using a single core of an Intel Core i7 processor with a clock speed of 2.6 GHz. No variance reduction techniques have been used.

Once a barrier transmission curve has been calculated (log *B* vs. thickness *t*), a fit to Equations (1) and (2) is performed to obtain values of TVL_1–3_ and TVL_1_ and TVL*_e_*. The Mathematica routine “NonlinearModelFit” has been used to find the best fit parameters to the logarithm of *B*. Monte Carlo–calculated statistical uncertainties have been used as input to this routine to find the total uncertainty (1 SD) in the values of TVL.

### 2.2 Tests of the code

Tests of the precursor skyshine MCLS code have been reported by McDermott.^16^ These tests include comparison with measurements made for the Kansas State University skyshine experiment in which the radiation had to traverse a 43 cm thick concrete roof and with the Los Alamos MCNP benchmark study.^22^, ^23^ The latter test involved comparison with MCNP, COG (a Boltzmann transport code), and measurements. The new tests of the MCBT code are described in the following paragraphs.

The first test is a low‐level test, in the limiting case of narrow beam geometry and monoenergetic radiation. Monoenergetic photons are all projected toward the barrier along the central axis of the beam (i.e., the field size is 0 × 0 cm^2^). Multiply scattered photons are killed. For this test case, the barrier transmission is defined as $B_{N}=N/N_{0}$, where *N* is the number of photons reaching the detector on the other side of the barrier, and *N*_0_ is the number of photons incident on the barrier. In this case, it is expected that $B_{N}=e^{-\mu t}$, where *μ* is the linear attenuation coefficient in concrete for the relevant beam energy, and *t* is the thickness of the concrete barrier. This has been tested for energies of 6.00 and 1.33 MeV. These energies correspond approximately to the average energy of the photons in an 18 and 4 MV beam, respectively. The thickness of the concrete ranges from 10 to 150 cm. The MC values have been corrected to account for air attenuation—which was present in an early version of the MCBT code. The photons traverse 6.3 m of air in reaching the detector. For 1.33 MeV, the air transmission is 0.965, and for 6 MeV, it is 0.984. The agreement between the MCBT values and $e^{-\mu t}$ has been evaluated by plotting the MCBT values of ln(*B_N_*) versus *t* and performing linear regression to find the slope of the line. The value of the slope should be the known value of *μ*. For the 6 MeV beam MC data, the slope of the line is 0.0634 ± 0.0002 cm^−1^ (95% confidence interval), whereas the actual value of *μ* is 0.0635 cm^−1^. For the 1.33 MeV beam, the slope of the line is 0.130 ± 0.0003 cm^−1^, and the value of *μ* is 0.130 cm^−1^.

In the second test, the incident beam is monoenergetic with a non‐divergent circular cross section. Multiple scattering is allowed, but only singly scattered photons are tallied. The only photons tallied at the detector are (1) primary photons that go right through without any scattering and (2) singly scattered photons. For a real barrier, under clinically realistic conditions, this is a poor assumption in that μt>>1, where *t* is the thickness of the barrier.

**FIGURE 1:**
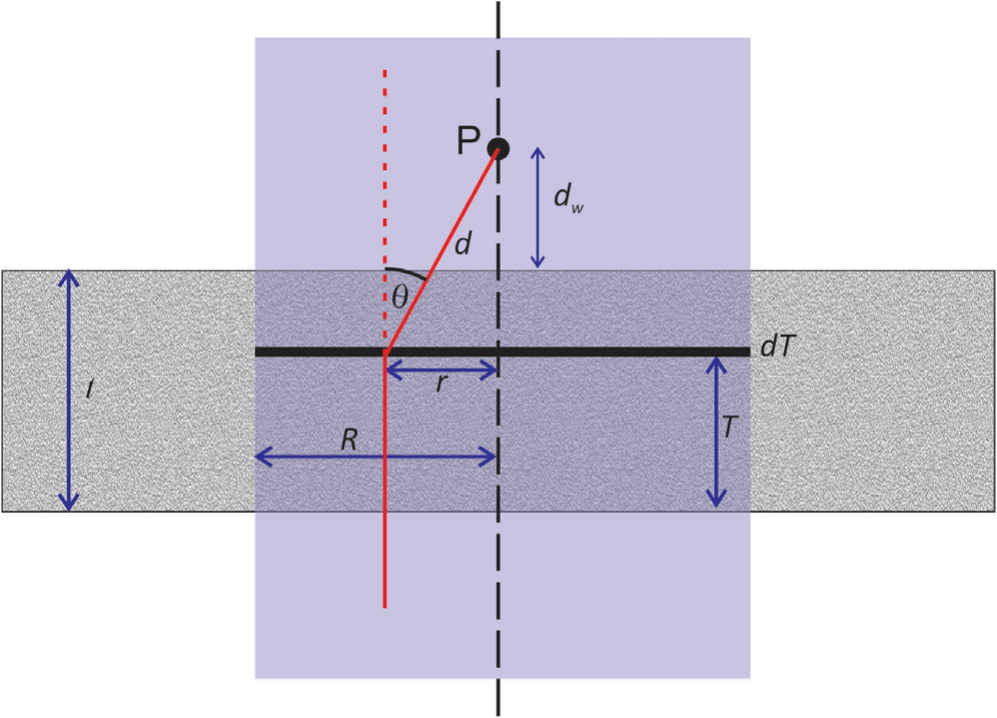
The geometry of the analytic test case for MCBT showing a cross section of a circular radiation field (shaded) with radius *R*, incident on a slab of concrete of thickness *t*. The point of “observation” is labeled P, and it is at a distance of *d_w_* from the distal face of the slab. The path of a representative photon is shown in red. This photon undergoes scattering at a depth *T* in the slab through an angle *θ*.

Under the assumptions described above, an analytic expression can be derived for the fluence reaching the detector. The geometry is shown in Figure 1. A non‐divergent radiation beam of circular cross section and radius *R* traverses a barrier of thickness *t*. A detector at point P is located at a distance of *d_w_* from the distal surface of the barrier. Consider a small volume element residing in the thin black layer in Figure 1. The number of photons scattered into solid angle ΔΩ by this volume element is given by

(5) $$\Delta N=\mathrm{dn}\Phi \left(T\right)\frac{d\sigma }{d\Omega }\Delta \Omega ,$$

where *dn* is the number of scattering centers, and Φ is the fluence, $\mathrm{dn}=\rho_{e}\mathrm{dV}$, where $\rho_{e}$ is the number of electrons per unit volume, $\Phi \left(T\right)=\Phi_{0}e^{-\mu T}$(Φ_0_ is the fluence incident on the barrier), dσ/dΩ is the is the Klein–Nishina differential cross section for Compton scattering, $\Delta \Omega =\Delta A/d^{2}$, ΔA is the area of the detector at point P, and *d* is the distance of the scattering element from point P. dV=2πrdrdT, $r=(d_{w}+t-T)\tan\theta$, and $d=(d_{w}+t-T)/\cos\theta$. The fluence arriving at a layer at depth *T* in the medium is assumed to be unscattered radiation only. This is consistent with the stipulation that only singly scattered photons reach the detector. A factor of $e^{-\mu^{\prime }\left(t-T\right)/\cos\theta }$ is inserted to account for the attenuation of the scattered photons, where $\mu^{\prime }$ is the linear attenuation coefficient of the scattered radiation. The fluence from singly scattered photons at the point P is:

(6) $$\begin{array}{c} d\Phi_{s}=\frac{\Delta N}{\Delta A}e^{-\mu^{\prime }\left(t-T\right)/\cos\theta }=\mathrm{dn}\Phi \left(T\right)\frac{d\sigma }{d\Omega }e^{-\mu^{\prime }\left(t-T\right)/\cos\theta }\frac{1}{d^{2}}. \end{array}$$

The barrier transmission is given by $B_{f}=\Phi_{t}/\Phi_{0}$, where $\Phi_{t}=\Phi_{p}+\Phi_{s}$, and $\Phi_{p}$ is the primary fluence (unscattered photons that traverse the barrier), $\Phi_{p}=\Phi_{0}e^{-\mu t}$. In this context, *B_f_* is the *fluence* transmission. An expression for the barrier transmission is obtained by assembling the various factors described above:

(7) $$\begin{array}{ccc} B_{f} & = & 2\pi \frac{\mu }{\sigma }\int_{0}^{t}e^{-\mu T}\left(\int_{0}^{\theta_{m}}e^{-\frac{\mu^{\prime }\left(\theta \right)\times \left(t-T\right)}{\cos\theta }}\tan\theta \frac{d\sigma }{d\Omega }d\theta \right)\mathrm{dT}\\ & & +\;e^{-\mu t}, \end{array}$$

where $\theta_{m}=\tan^{-1}\frac{R}{d_{w}+t-T}$, and $\mu =\sigma \rho_{e}$(*σ* is the total cross section per electron).

Some physical insight into the general problem of barrier transmission can be obtained by considering Equation (7) in the limit of small angle scattering even though this is not the prevailing condition for shielding evaluation, except for very small fields. This limit provides a first‐order correction to narrow‐beam attenuation. We expand the inner integrand in Equation (7) to leading order in *θ*. For $d_{w}\gg R$ and $d_{w}\gg t$ (implies $\theta_{m}\ll 1$, small angle scattering):

(8) $$e^{-\frac{\mu^{\prime }\left(\theta \right)\times \left(t-T\right)}{\cos\theta }}\tan\theta \frac{d\sigma }{d\Omega }\approx e^{-\mu (t-T)}\theta r_{0}^{2},$$

where *r*_0_ is the classical electron radius. Equation (8) is substituted into Equation (7) to obtain an expression for narrow‐beam transmission:

(9) $$B\approx e^{-\mu t}\left\{1+\pi \frac{r_{0}^{2}}{\sigma }\mu t{\left(\frac{R}{d_{w}}\right)}^{2}\right\}.$$

The term in curly brackets is the buildup factor. The scatter component depends on $\pi R^{2}$ as expected, and this term is inversely proportional to $1/d_{w}^{2}$—inverse square of the distance from the distal surface of the barrier. Equation (9) is not expected to be even approximately valid in the broad‐beam case because $d_{w}\ll R$.

**FIGURE 2:**
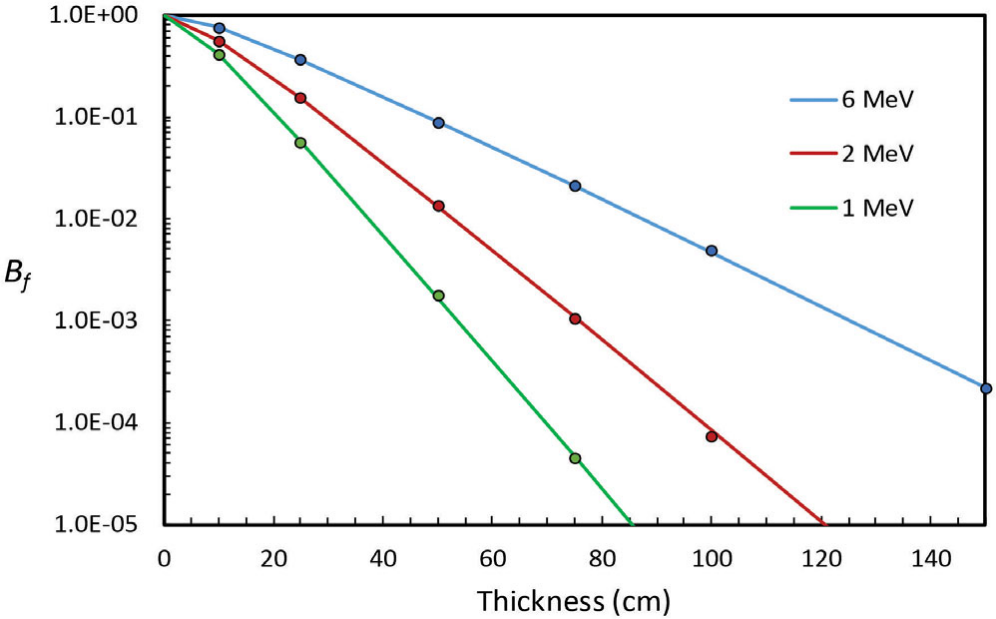
This graph shows the results of a test of the MCBT code for three monoenergetic broad beams with circular cross section (radius = 1.35 m). The quantity *B_f_* is the fluence transmission. Multiple scattering is permitted, but only singly scattered photons are tallied. The solid curves represent numerical evaluation of the analytic expression for *B* from Equation (7). The data points are the result of MCBT calculations.

The MCBT code has been tested against Equation (7) by using monoenergetic photons that are projected toward the barrier as a plane parallel beam of circular cross section with no divergence and a radius of 1.35 m. This radius corresponds to the beam radius at a distance of 6.0 m from the source of a diverging beam with an opening half angle of 12.7°. The distance *d_w_* has been set to 0.3 m. In the MCBT code, only photons that have scattered once, or not at all, are tallied. Equation (7) assumes that all of the interactions that occur at the point of scattering are Compton. For this reason, photoelectric and pair productions are turned off in the MCBT code for this test. This makes about a 10% difference in the calculated *B_f_*. The test was carried out for energies of 1, 2, and 6 MeV and for thicknesses ranging from 10 to 150 cm. The results are shown in Figure 2, which displays a graph of *B_f_* versus barrier thickness. The solid curves are the result of numerical integration of Equation (7), and the individual points are from MCBT calculations. The average values of the ratio of *B_f_* from MCBT to that from Equation (7) are 1.00, 0.97, and 1.00 for the 1, 2, and 6 MeV case, respectively. The coefficient of variation for the MCBT points in Figure 2 is about 3%–5%.

## 3 RESULTS

### 3.1 Barrier transmission and TVL

**FIGURE 3:**
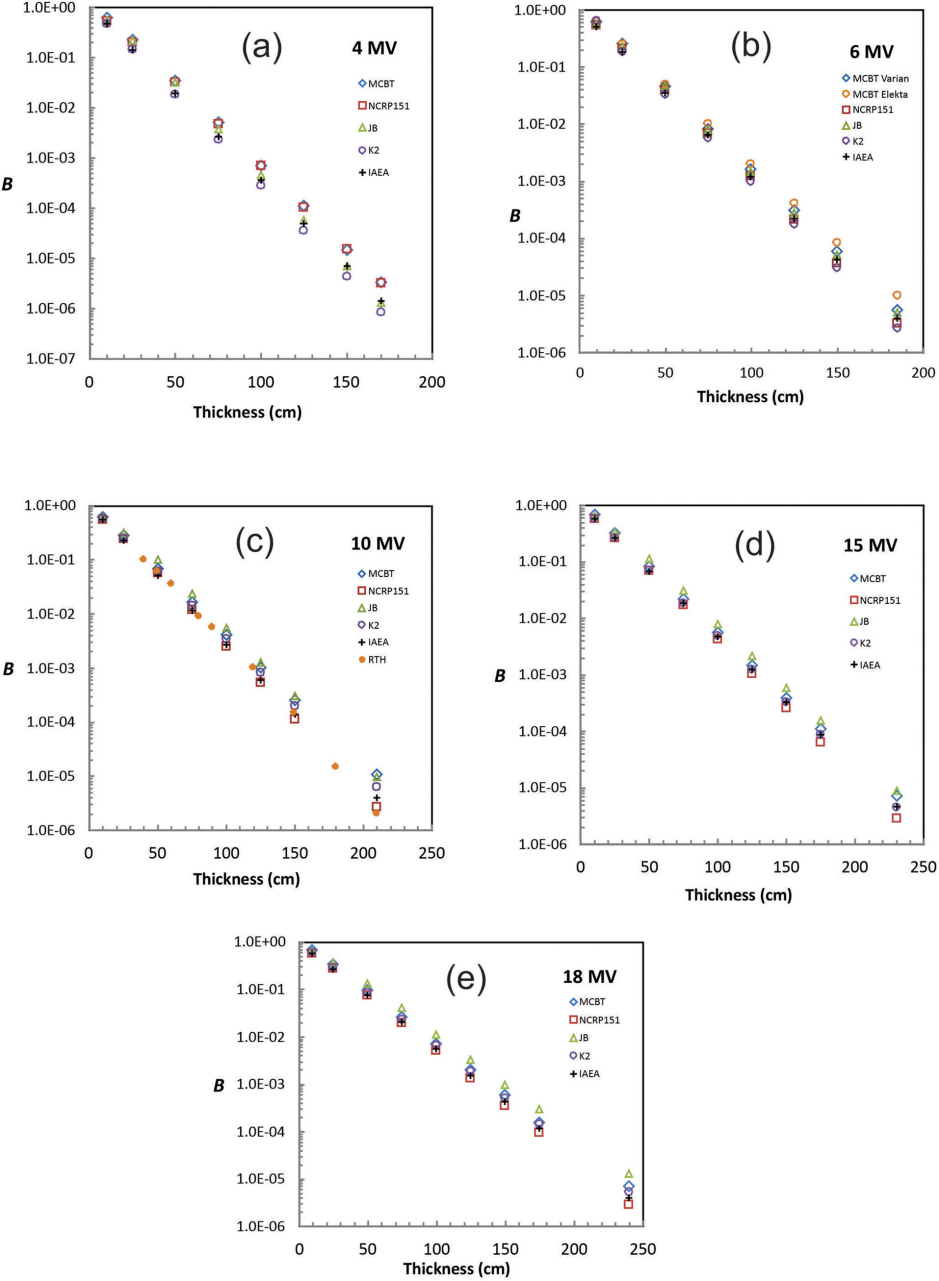
Barrier transmission, *B*, versus thickness for (a) 4 MV, (b) 6 MV, (c) 10 MV, (d) 15 MV, and (e) 18 MV. Values of *B* for National Council on Radiation Protection and Measurements Report No. 151 (NCRP151), Jaradat and Biggs (JB), Karoui and Kharrati (K2), and International Atomic Energy Agency (IAEA) (and Rijken, Town, and Healy [RTH] for 10 MV) are also shown for comparison. The spread in the values of *B* increases with increasing thickness. The MCBT values of *B* are in good agreement with NCRP151 for 4 MV but are substantially larger for all other energies.

Barrier transmission values have been computed using the MCBT code for beam energies of 4, 6, 10, 15, and 18 MV. The nominal distance to the distal surface of the barrier is 6.0 m from the linac target. The nominal field size at the isocenter is 40 × 40 cm^2^. Values of *B* are for a distance of 0.3 m from the distal barrier surface only. Graphs of log *B* versus thickness are shown in Figure 3a–e. Error bars for MCBT are not included in these log plots because they are generally too small to see. The standard deviation in the values of *B* is less than 10% for the thickest barriers studied and much less than this for barriers less than 50 cm thick. Values of *B* for NCRP151, JB, K2, IAEA, and RTH (for 10 MV only) are also shown in Figure 3 for comparison. These values are based on the TVL data from those references. For all energies, the spread among the values of *B* increases with increasing thickness. For *t* > TVL_1_, the slope of the log *B* versus *t* plot is 1/TVL*_e_* (see Equation 1). Small differences in TVL*_e_* result in large differences in log *B* (and much larger differences in *B* itself) when the line is extended to thick barriers.

For 4 MV, the MCBT values are in good agreement with NCRP151, generally within about 10%. This is surprising in that the 4 MV NCRP151 values are based on an extrapolation from other energies. For energies other than 4 MV, NCRP151 values of *B* are as much as a factor of 3 lower than MCBT. For 6 MV, the MCBT values of *B* are higher than all the other values, by up to a factor of 2. The 6 MV Varian values of *B* are in best agreement with JB differing by less than 15%. The Elekta 6 MV MCBT values of *B* are larger than those for Varian by a factor of 1.6 for a thickness of 180 cm. This is expected based on differences in the energy of the incident beam as discussed in Section 2.1. Discrepancies between measurements and NCRP151 predictions are expected to be greater for 6 MV Elekta linacs than Varian linacs.

For 10 MV, there is a large spread in the values of *B*. The MCBT values are up to a factor of 4 larger than NCRP151 and up to a factor of 5 larger than RTH for a barrier thickness of 210 cm. The agreement with JB is best—it is within about 20%. The TVL*_e_* value quoted by Martin and McGinley (38.9 cm) is the same as quoted by IAEA and similar to the K2 value (40.2 cm). RTH quote a single value of the TVL for their Monte Carlo barrier transmission curve, yet this curve clearly changes slope with increasing thickness. The RTH value of *B* is actually lower than predicted by NCRP at a thickness of about 200 cm, although the error bar is quite large.

For 15 MV, *B* is up to a factor of 2.5 times that of NCRP151. In this case, the agreement with JB is the best. For 18 MV, agreement with K2 is within about 15%, whereas MCBT *B* values are up to a factor of 1.6 times larger than NCRP values. All of these comparisons would be of less concern were it not for the fact that for energies greater than 4 MV, NCRP151 underestimates predicted AKR by relatively large factors.

Table 4 lists the best fit MCBT computed values of TVL_1–3_ (based on Equation 2) and TVL_1_ and TVL*_e_* (based on Equation 1). Statistical errors are in parentheses (1 SD). The relative residual shown in Table 4 is the average value of $|B\left(t_{i}\right)-B_{i}|/B_{i}$, where *B*(*t_i_*) is the calculated value from either Equation (1) or Equation (2) based on the best fit values, and *B_i_* is the actual MCBT‐calculated value for thickness *t_i_*. Examination of the table shows that the expected statistical error in the values of *B* calculated from the TVL is on the order of 5% for TVL_1–3_ and 10% for TVL_1_ and TVL*_e_*. The TVL values for NCRP151, JB, K2, and IAEA are listed in the paper by McDermott et al.^3^

**TABLE 4:** Tenth value layer (TVL) data for concrete barriers^a^

Energy (MV)	TVL_1_ (cm)	TVL_2_ (cm)	TVL_3_ (cm)	Relative residual (%)	TVL_1_ (cm)	TVL*_e_* (cm)	Relative residual (%)
4	38.7 (±0.4)	26.8 (±0.5)	29.8 (±0.2)	5.8	37.4 (±0.3)	29.2 (±0.1)	6.6
6	42.5 (±0.4)	29.6 (±0.6)	35.1 (±0.4)	4.9	41.2 (±0.3)	33.2 (±0.2)	9.8
6^b^	41.7 (±0.4)	32.7 (±0.6)	36.6 (±0.4)	3.4	40.7 (±0.3)	35.1 (±0.2)	7.9
10	44.7 (±0.3)	39.0 (±0.4)	42.3 (±0.4)	3.2	44.0 (±0.2)	40.9 (±0.2)	5.4
15	49.2 (±0.4)	39.1 (±0.7)	44.6 (±0.4)	5.1	48.3 (±0.3)	42.6 (±0.2)	9.2
18	51.5 (±0.4)	41.3 (±0.7)	46.3 (±0.4)	5.6	50.4 (±0.3)	44.6 (±0.2)	7.1

*Note*: TVL_1–3_ are based on the best fit to Equation (2), and TVL_1_ and TVL*_e_* are based on the best fit to Equation (1). Errors are ±1 standard deviation.

^a^ Side length of the field = 240 cm at the distal surface of the barrier.

^b^ Elekta beam spectrum.

It is not surprising that the fits to the MCBT‐calculated values of *B* are somewhat better for the three parameter fit to TVL_1–3_ based on Equation (2) versus the two parameter fit (TVL_1_ and TVL*_e_*) based on Equation (1). This is shown by the relative residual values. The three parameter fits, however, are not much better, and it would seem that the two parameter fit using TVL_1_ and TVL*_e_* does not result in a significant loss in accuracy.

Comparison of calculated values of *B* with measurements is complicated by the many, often uncontrolled factors, such as concrete density, field size *at the barrier*, distance from the distal surface, and additional materials on the barrier, such as steel laser mounting plates or a façade of masonry. One such comparison can be made here. This was first considered by McDermott et al. for an Elekta linac.^3^ The density of the concrete is known (2.29 g/cm^3^), the thickness of the barrier was verified by measurement, and the MU rate of the linac was measured. The only materials unaccounted for in the barrier are rebar, dry wall, and an aluminum laser mounting plate. The thickness of the barrier is 183 cm, the distance from the target to the distal surface is 8.0 m, and the linac has energies of 6, 10, and 15 MV. Table 5 shows the ratios of the measured AKR versus that predicted using various values of the TVL. The TVL values have been multiplied by 2.29/2.35 = 0.974 to account for the measured density. Measured values are approximately a factor of 2 greater than predicted by NCRP151 and slightly less than predicted by MCBT.

**TABLE 5:** Comparison between predicted and measured air kerma rate (AKR) for a single barrier

Energy (MV)	Measured/NCRP	Measured/JB	Measured/K2	Measured/MCBT
6^a^	2.04	1.63	2.48	0.74
10	2.22	0.75	1.10	0.75
15	1.78	0.86	1.30	0.98

Abbreviation: JB, Jaradat and Biggs; K2, Karoui and Kharrati

^a^ Elekta beam spectrum.

### 3.2 Energy of transmitted photons

**FIGURE 4:**
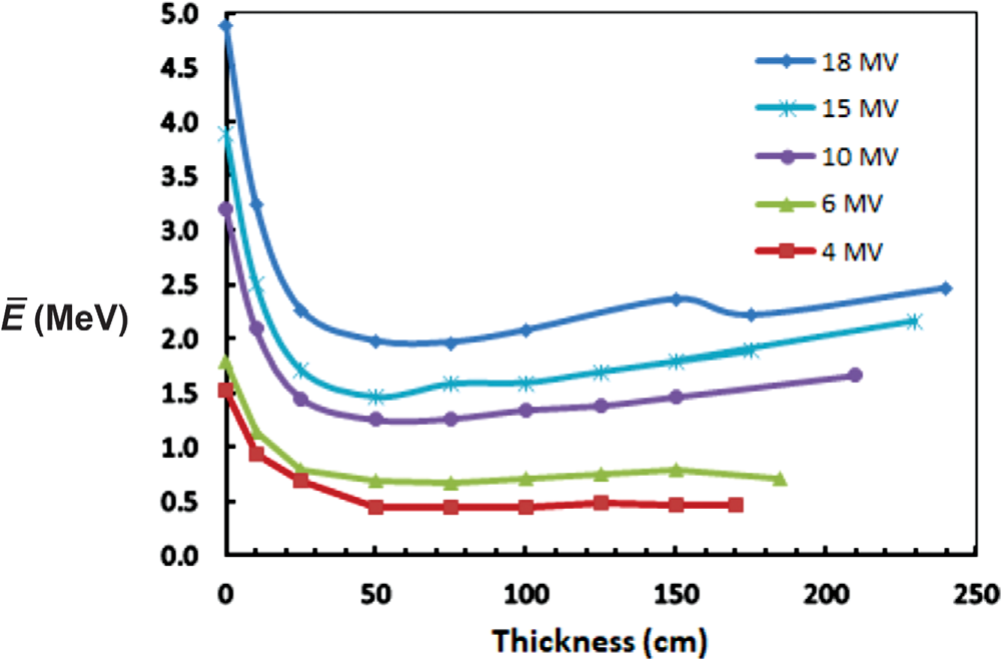
The average energy of the emergent spectrum as a function of the thickness traversed. The energy of all beams drops precipitously in the first 20–30 cm. The average energy reaches a minimum at a thickness of about 50 cm, rising slowly as the thickness increases beyond this.

Figure 4 shows the average energy of the emergent beam as a function of barrier thickness. The average energy drops precipitously as the thickness increases from 0 to 50 cm, where it reaches a minimum value for all energies and then slowly increases with increasing thickness thereafter. The beams are “softened” initially, not hardened. This is consistent with the TVL_2_ values in Table 3: TVL_2_ is less than TVL_1_ for all beam energies. This is due to the dominance of scattered photons. As an example, for 10 MV and *t* = 45 cm ∼ TVL_1_, (field size 40 × 40 cm^2^, *d_s_* = 6 m), the number of unscattered primary photons reaching the detector is only about 10% of all photons reaching the detector. This contribution to the spectrum is undoubtedly hardened, but this is dominated by the other 90% of the photons that are either singly or multiply scattered. For *t* > 50 cm, the beam energy increases slowly, and TVL_3_ is always larger than TVL_2_. The energy distribution strongly peaks at energies less than about 200 keV. When the energy of the photons becomes $E_{\gamma }\ll m_{0}c^{2}$ (rest mass energy of the electron), Compton scattering transitions to Thomson scattering. In this limit, the photons lose very little energy as they scatter. The Compton and the photoelectric cross section are equal at about 55 keV for concrete, and for energies below this the photoelectric effect rapidly becomes much larger than Compton as the energy decreases. Thus, when photon energy declines below 55 keV, photons are strongly absorbed. This may explain the slight increase in average energy that occurs for *t* > 50 cm—low‐energy photons are removed by photoelectric, leaving higher energy scattered photons.

The pronounced decrease in the average energy of the photons emerging from the barrier implies that any additional shielding or extraneous material, such as steel or lead, placed on the outside of the barrier is likely to have a much greater shielding effect than otherwise expected.

### 3.3 Distance and field size dependence of air kerma rate

When the barrier is especially thin or the field size is very small, an inverse square dependence of the AKR based on a point source at the linac target is fairly accurate. The equation *P* = *B*(0.3) WUT/*d* ^2^ (where *B* is determined at 0.3 m distal to the barrier) is invalid for distances beyond the distal surface of the barrier under most circumstances. Multiple scattering is so dominant that the barrier appears to become a new source of radiation and this source is not a point source, except for exceedingly large distances. The AKR beyond the point of measurement at 0.3 m from the barrier is significantly higher than an application of the inverse square law would suggest. This has implications for high occupancy areas beyond the immediate proximity of the barrier. For a distal barrier surface at 6 m from the target, the AKR is more than a factor of 2 larger at a distance of 20 m from the source than predicted by the inverse square law (assuming that the source is at the target of the linac).

An isotropic circular source having a radius *R* produces an intensity that varies along an axis through the center as

(10) $$P\propto \ln\left[1+{\left(\frac{R}{d-d_{s}}\right)}^{2}\right],$$

where *R* is the field size at the barrier, and *d_s_* is the distance from the X‐ray target to the distal surface of the barrier. At large distance, when $R/(d-d_{s})\ll 1$, $P\propto {\left[R/\left(d-d_{s}\right)\right]}^{2}$. There is no reason to assume that the transmitted radiation emerges isotropically, but we can still fit the calculated AKR to Equation (10) by assuming an effective value of *R*. The AKR at distances *d* > *d_s_* + *d_w_* can be expressed as

(11) $$P\left(d\right)=B\left(d_{w}\right)\frac{\mathrm{WUT}}{{\left(d_{s}+d_{w}\right)}^{2}}\frac{\ln\left[1+{\left(\frac{\alpha (f+f_{0})}{d-d_{s}}\right)}^{2}\right]}{\ln\left[1+{\left(\frac{\alpha (2.4+f_{0})}{d_{w}}\right)}^{2}\right]},$$

where *B*(*d_w_*) is the barrier transmission for a field size at the barrier of 2.4 m, *f* is the field size at the barrier, *α* is a fitting constant, and *f*_0_ is an offset fitting parameter. Even when *f* = 0, there is some spreading of the beam due to scattering, and this may explain the offset term *f*_0_. When $d\gg d_{s}$, *P*(*d*) is proportional to 1/*d* ^2^. The values of *α* and *f*_0_ depend on the thickness of the barrier and energy. Optimal values for all energies and barrier thicknesses (provided the barrier is thick) are roughly *α* = 28 and *f*_0_ = 0.02 m, respectively.

**FIGURE 5:**
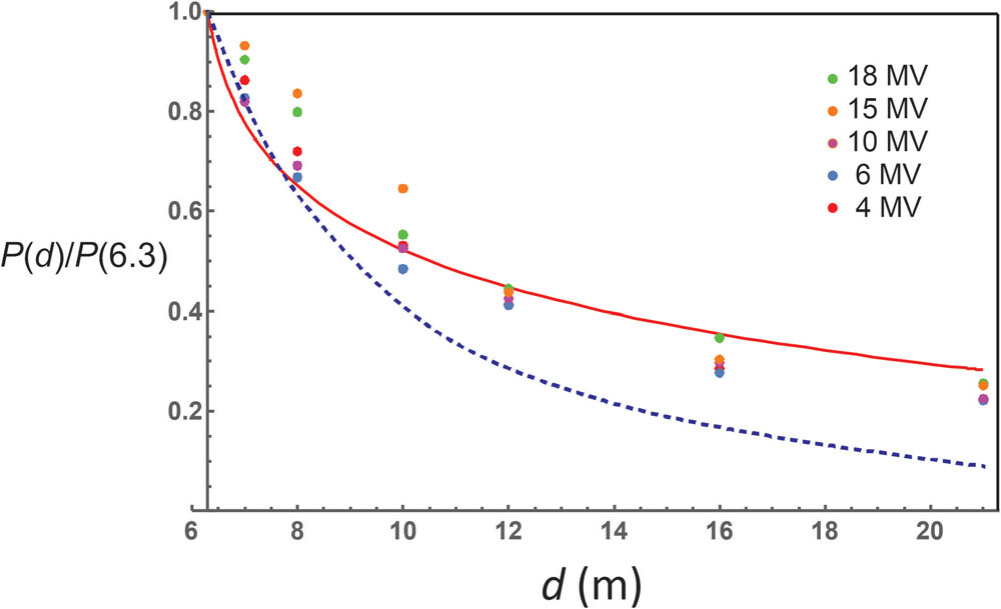
Distance dependence (from the linac target) of the transmitted radiation along the central axis for field size of 2.4 m and *d_s_* = 6.0 m. The air kerma rate is normalized to 1.0 at *d* = 6.3 m. Also shown is the inverse square dependence assuming the source is at the linac target (dashed curve). The air kerma rate (AKR) falls off more slowly than inverse square and is significantly higher at a distance of 20 m than a simple inverse square law would predict. The red solid curve is the graph of Equation (11) with fitting parameters *α* = 28 and *f*_0_ = 0.02 m.

Figure 5 shows the relative distance dependence of the AKR for *d* > *d_s_* = 6.0 m for all energies and for a 240 cm field size at the barrier. The AKR is normalized to a value of 1.0 at *d* = 6.3 m. The barrier thicknesses vary from 1.5 m for 4 MV to 2.4 m for 18 MV. The data points in this graph are the MCBT results. The dashed curve is an inverse square law assuming the source of the radiation is at the linac target. The solid curve is based on Equation (11) with values *α* = 28 and *f*_0_ = 0.02 m. The best fit value of the fitting constants *α* and *f*_0_ depend on energy and barrier thickness, but Equation (11) is not very sensitive to the values. The new “pseudo source” created by scattering from the barrier acts as if it is a very large isotropic radiator. The effective size of the source grows as the barrier thickness increases. Inverse square behavior is not expected until *d* > 50 m.

**FIGURE 6:**
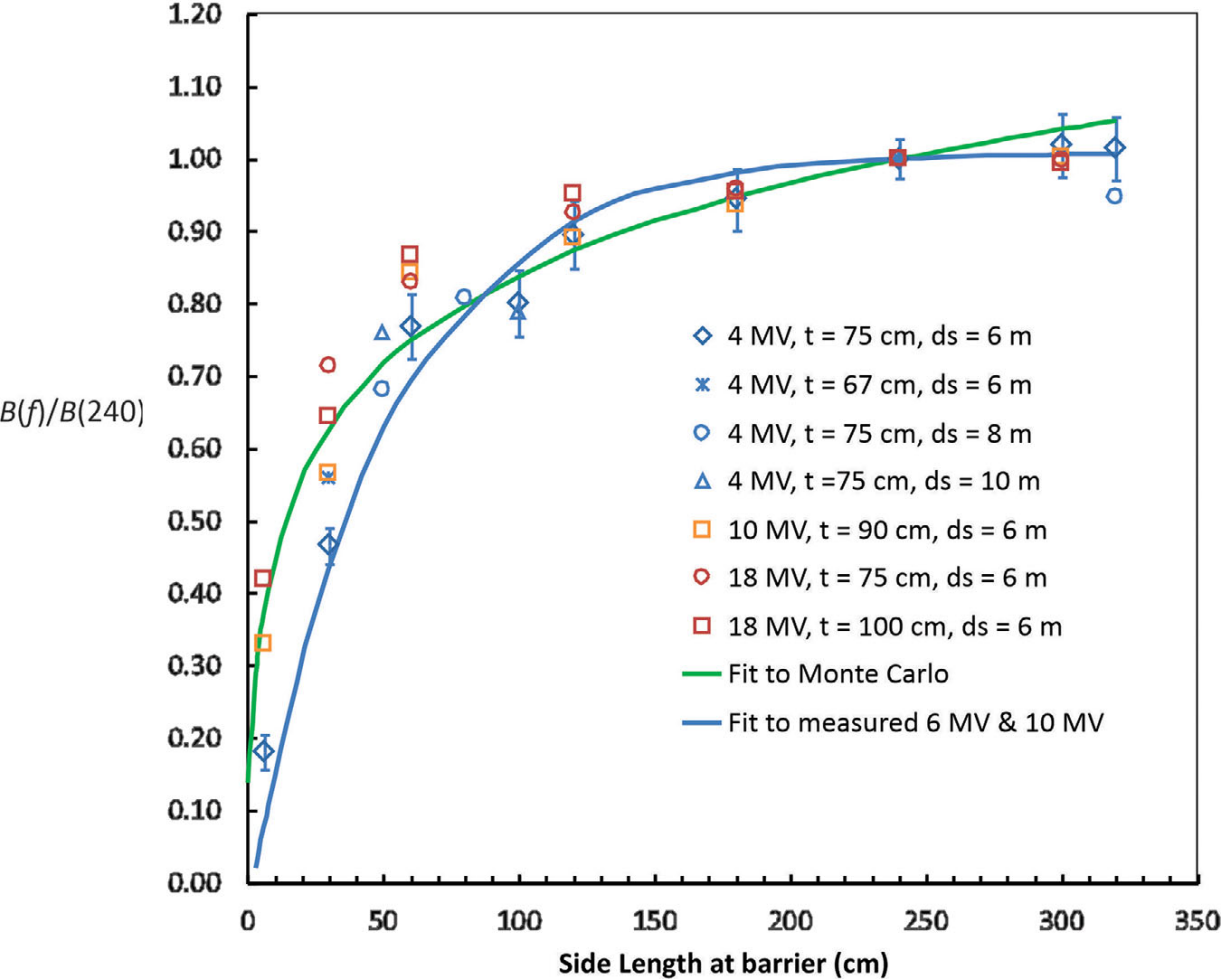
Relative field size dependence of the barrier transmission for 4, 10, and 18 MV and a variety of thicknesses and distances to the distal barrier surface (*d_s_*) as a function of the field size at the distal surface. All values are normalized to 1.0 for a field size of 240 cm (40 cm at isocenter for *d_s_* = 6 m). The 4 MV beams show the greatest field size dependence. Error bars are shown for one data set. The green curve is the fitting function given by Equation (11) with fitting parameters *α* = 28 and *f*_0_ = 0.02 m. The blue curve is from measurements for 6 and 10 MV.

The field size dependence of *B* is shown in Figure 6 for energies of 4, 10, and 18 MV. The graph shows the relative dependence of *B* on the side length *f* of the square field at the distal surface of the barrier. The graph also shows a fit to measured values of the relative barrier transmission (blue curve) from McDermott et al. for 6 and 10 MV.^10^ The values are normalized to 1.0 for *f* = 240 cm. A side length of 40 cm at isocenter diverges to 240 cm at a distance of 6.0 m. The value of *B* is most sensitive to field size for an energy of 4 MV. The barrier transmission for a 60 cm field is about 80% of that for a 240 cm field. The field size dependence saturates for fields larger than about 150 cm in side length. When the field becomes larger than this, additional scattered photons may be unable to reach the central axis at *d_w_* = 0.3 m. The green curve in Figure 6 is a graph of Equation (11) with parameters *α* = 28 and *f*_0_ = 0.02 m. The Monte Carlo results shown in Figure 6 do not account for any off‐axis change in the photon energy.

## 4 CONCLUSIONS

There are both experimental and theoretical reasons to believe that the TVL data in references NCRP151, IPEM75, and IAEA significantly underestimate air kerma rates outside concrete primary barriers. A new set of TVL data for linac primary concrete barriers is presented here for beam energies of 4, 6, 10, 15, and 18 MV. These values are listed in Table 4. Comparisons with previous determinations can be seen in Figure 3. The TVL calculations have been done using the Monte Carlo method and a definition of the transmission that is mathematically consistent with its use in standard shielding calculations (see Equation 3). Beam spectra for contemporary linacs have been used that reproduce measured depth–dose curves very accurately. The calculations have been extended to thicknesses that are clinically realistic.

Transmission depends on the field size at the barrier. The entries in Table 4 are for a field length of 240 cm at the distal surface of the barrier. Fortunately, *B* is not very sensitive to field size, for field sizes >150 cm. This is shown in Figure 6. The average energy of the transmitted radiation is considerably lower than the incident energy and strongly peaks at energies less than about 200 keV. The energy reaches a minimum for all initial beam energies at a thickness of about 50 cm and rises slowly with increasing thickness thereafter. The radiation beams are initially softened, not hardened. This is illustrated in Figure 4. The air kerma rate at distances beyond 0.3 m from the distal surface of the barrier is significantly underestimated by an inverse square dependence based on the distance from the linac target. It is as if there is a new source of radiation emanating from the distal surface of the barrier, and this source is effectively large and therefore not a point source. This may be important in the case in which there is a high occupancy area beyond a low occupancy area that is immediately contiguous to the barrier.

## CONFLICT OF INTEREST

The authors have no relevant conflicts of interest to disclose.

## ENDNOTES

* Elekta: Versa HD Product Data, undated.

† The symbol “∼” means order of magnitude.

‡ The Varian “Designers Desk Reference” (Vol. 16, No. 4, 2016).

# Elekta Oncology Products “Site Planning Guide” (2019).
